# Brain Mass and Encephalization Quotients in the Domestic Industrial Pig (*Sus scrofa*)

**DOI:** 10.1371/journal.pone.0157378

**Published:** 2016-06-28

**Authors:** Serena Minervini, Gianluca Accogli, Andrea Pirone, Jean-Marie Graïc, Bruno Cozzi, Salvatore Desantis

**Affiliations:** 1 Section of Veterinary Clinics and Animal Productions, Department of Emergency and Organ Transplantation (DETO), University of Bari Aldo Moro, Valenzano (Ba), Italy; 2 Department of Veterinary Sciences, University of Pisa, Pisa, Italy; 3 Department of Comparative Biomedicine and Food Science, University of Padova, Padova, Italy; University of Lethbridge, CANADA

## Abstract

In the present study we examined the brain of fetal, newborn, and adult pigs raised for meat production. The fresh and formalin-fixed weights of the brain have been recorded and used, together with body weight, to calculate the Encephalization Quotient (EQ). The weight of the cerebellum has been used to calculate the Cerebellar Quotient (CQ). The results have been discussed together with analogue data obtained in other terrestrial Cetartiodactyla (including the domestic bovine, sheep, goat, and camel), domesticated Carnivora, Proboscidata, and Primates. Our study, based on a relatively large experimental series, corrects former observations present in the literature based on smaller samples, and emphasizes that the domestic pig has a small brain relative to its body size (EQ = 0.38 for adults), possibly due to factors linked to the necessity of meat production and improved body weight. Comparison with other terrestrial Cetartiodactyla indicates a similar trend for all domesticated species.

## Introduction

The pig (*Sus scrofa domesticus*) is one of the first domesticated mammals, and represents a very diffuse, important and traditional meat resource in many countries, in which millions of individuals of the species are raised and slaughtered for production. The pig is also a useful experimental model in different areas of biomedical research world-wide.

Although several textbooks addressed the anatomy of the pig in detail [1–4], an original, exhaustive and functional description of the brain of this species is lacking. Data on the weight of the brain of the pig and relative brain to body weight ratio are reported in Table 1, with the relative relevant literature and reference texts.

**Table 1:** Data from literature rative to the ratio brain/body weight of the swine.

Year	Source	Brain weight (g)	Body weight (kg)	Ratio
1879	[57]	160		
1912	[58]	125–164, *domestic*	250*	1:2000–1:9000
		178, *wild*		
1913	[59]	112		
1927	[60]	112, *adult*		
		14, *neonate*		
1909	[37]	162	157,5	1:972
			74	1:705
1969	[61]	180		
1988	[18]	96–145	60–96	1:630–1:660
		105–110	126–209	1:1200–1:1900
2005	[21]	259	89,4	
2006	[4]	111–123*	80–90	1:650
			200	1:1800
2007	[53]	95,3		
2010	[27]	180	125	
2012	[15]	169.8	867,72	
		70,2	22,15	
		60,6	27,7	
		57,6	27,5	
		47,7	10	
		28,8	0.478	
2012	[16]	95,3		
2013	[55]	95,3		

*estimate

There is a growing awareness of consumers and of the general public towards animal welfare, and the current European legislation includes several measures to minimize animal stress in the production farms and also during transportation and at the slaughterhouse. The behavior of the domestic swine has been actively investigated considering maternal-neonatal interactions [5], disease [6], feeding [7], aggression and affiliation during social conflict [8], and during group housing [9]. However, identification of the associative areas of this species and the functional wiring of the relative circuitry is still poorly understood. Under these conditions the neural foundation of behavior, motivation and social interaction are only tentatively identified based on comparative studies performed in rodents, primates or other mammals.

The encephalization quotient (EQ), defined as the ratio between observed and expected brain mass [10,11], is a parameter widely applied in comparative mammalian neuroanatomy. The EQ value indicates whether a species possesses a brain larger (EQ > 1), equal (EQ = 1) or smaller (EQ <1) than expected for its body mass. The cerebellar quotient (CQ) is a similar parameter that assesses the relative development of the cerebellum.

In this study we considered the EQ and CQ of the adult domestic pig and compared the results to those obtained in other mammalian species, with a special attention to additional members of the order Cetartiodactyla. Furthermore, we investigated the change of EQ, CQ, and weight of the single cerebral vesicles from newborn to adult state. Since the most diffuse pig breed in the industrial world is the pure or cross-breed Landrace (LR) pig of Danish origin, we focused our investigation on this specific variety together with the similar and also very diffuse Large White (LW) pig, as they represent the “type” of animal most commonly found in commercial intensive farming.

## Materials and Methods

### Brain sampling

For the present study we used brains from 48 *Sus scrofa domesticus* (31 adults, 13 newborns, 4 fetuses, males and females, see Table 2). The animals were LR or LW breeds, or mixed LR x LW. Pure of cross-bred LR pigs include more than 90% of all pigs commercially raised in the Western world. The LW breed is the other popular breed used in animal production all over the world. The two breeds have the same average body weight at the age considered here.

**Table 2:** Details of the animals used in the experimental series.

Sample	Date of slaugther	Breed	Age (days)	Maturity	Body weight (kg)	Brain weight (g)	Sex
1	29/06/2013	Landrace (LR)	1	Neonate	1.100	30	
2	29/06/2013	LR	1	Neonate	1.280	33	
3	29/06/2013	LR	1	Neonate	1.100	32.91	M
4	29/06/2013	LR	1	Neonate	1.750	32.26	M
5	09/07/2013	Large White (LW)	300–360	Adult	190–200	148.53	F
6	09/07/2013	LW	300–360	Adult	190–200	115.54	F
7	09/07/2013	LW	300–360	Adult	190–200	136.08	F
8	09/07/2013	LW	300–360	Adult	190–200		F
9	14/07/2013	LR	1	Neonate	1.120	31.98	M
10	14/07/2013	LR	1	Neonate	0.900	29.8	M
11	14/07/2013	LR	1	Neonate	0.940	28.84	F
12	14/07/2013	LR	1	Neonate	1.230	34.06	F
13	14/07/2013	LR	1	Neonate	1.530	35.5	M
14	23/07/2013	LW	180–240	Young adult	74–80	116.1	F
15	23/07/2013	LW	180–240	Young adult	74–80	128.15	F
16	23/07/2013	LW	180–240	Young adult	74–80	134.39	F
17	23/07/2013	LW	180–240	Young adult	74–80		F
18	23/07/2013	LW	G 92–96	Fetus	0.12	12	M
19	23/07/2013	LW	G 92–96	Fetus	0.16	16	F
20	23/07/2013	LW	G 92–96	Fetus	0.167	16.7	M
21	23/07/2013	LW	G 92–96	Fetus	0.148	14.75	F
22	08/10/2013	LW	240–300	Adult	150–160	122.42	F
23	08/10/2013	LW	240–300	Adult	150–160	136.7	F
24	08/10/2013	LW	240–300	Adult	150–160	128.86	F
25	08/10/2013	LW	240–300	Adult	150–160	138.27	F
26	28/10/2013	LR	1	Neonate	0.850	31.17	F
27	28/10/2013	LR	1	Neonate	0.870	33.54	M
28	04/11/2013	LR	1	Neonate	1.350	34.19	F
29	04/11/2013	LR	1	Neonate	1.340	35.11	M
30	13/11/2013	LR x LW	240–300	Adult	130–150	131.32	M
31	13/11/2013	LR x LW	240–300	Adult	130–150	139.41	F
32	13/11/2013	LR x LW	240–300	Adult	130–150	120.72	M
33	13/11/2013	LR x LW	240–300	Adult	130–150	146.39	F
34	13/11/2013	LR x LW	240–300	Adult	130–150	140.15	F
35	13/11/2013	LR x LW	240–300	Adult	130–150	151.5	M
36	13/11/2013	LR x LW	240–300	Adult	130–150	125.98	F
37	13/11/2013	LR x LW	240–300	Adult	130–150	147.48	F
38	04/12/2013	LR x LW	240–300	Adult	190–200	133.78	M
39	04/12/2013	LR x LW	240–300	Adult	190–200	160.26	F
40	04/12/2013	LR x LW	240–300	Adult	190–200	139.65	F
41	04/12/2013	LR x LW	240–300	Adult	190–200	140.53	M
42	04/12/2013	LR x LW	240–300	Adult	130–150	118.93	F
43	04/12/2013	LR x LW	240–300	Adult	130–150	138.54	F
44	04/12/2013	LR x LW	240–300	Adult	130–150	126.77	M
45	04/12/2013	LR x LW	240–300	Adult	130–150	107.08	F
46	04/12/2013	LR x LW	240–300	Adult	130–150	122.35	F
47	04/12/2013	LR x LW	240–300	Adult	130–150	134.04	F
48	04/12/2013	LR x LW	240–300	Adult	130–150	136.58	M

G: gestational age in days (duration of the pregnancy is approx. 114 days).

The brain of the adults was removed at the slaughterhouse (Maselli Industrie Srl 41°7’9.956” N; 16° 28’29.975” E), where animals were treated according to the European Community Council Regulation (CE1099/2009) concerning animal welfare during the commercial slaughtering process, and were constantly monitored under mandatory official veterinary medical care. All the animals considered here were in good body condition and considered free of pathologies by the veterinary medical officer responsible for the health and hygiene of the slaughterhouse. Adult carcasses were put on the market, while newborns and fetuses were discarded in accordance with the Regulation CE 1069/2009.

Four fetuses, born from the same sow whose pregnancy was undetected prior to slaughtering, were collected at the slaughterhouse and transported to the necropsy room of the Veterinary Clinic and Animal Productions Section, Department of Emergency and Organ Transplantation, of the University of Bari “Aldo Moro”, Italy. There the fetal brains were removed and weighted. Samples from newborns animals were collected in the latter location from individuals who did not survive after birth and whose death resulted unrelated to the nervous system.

The body weight of adult was determined for each animal by the staff of the abattoir, whereas the weight of fetuses and newborns was measured in the necropsy room. The age of the fetuses was determined based on references in literature using cranium-sacrum distance [12]. All brains were extracted in 3-48h *post mortem*, and if extraction was not possible during the day, samples were kept at 4°C.

### Determination of brain weight

**Fig 1:**
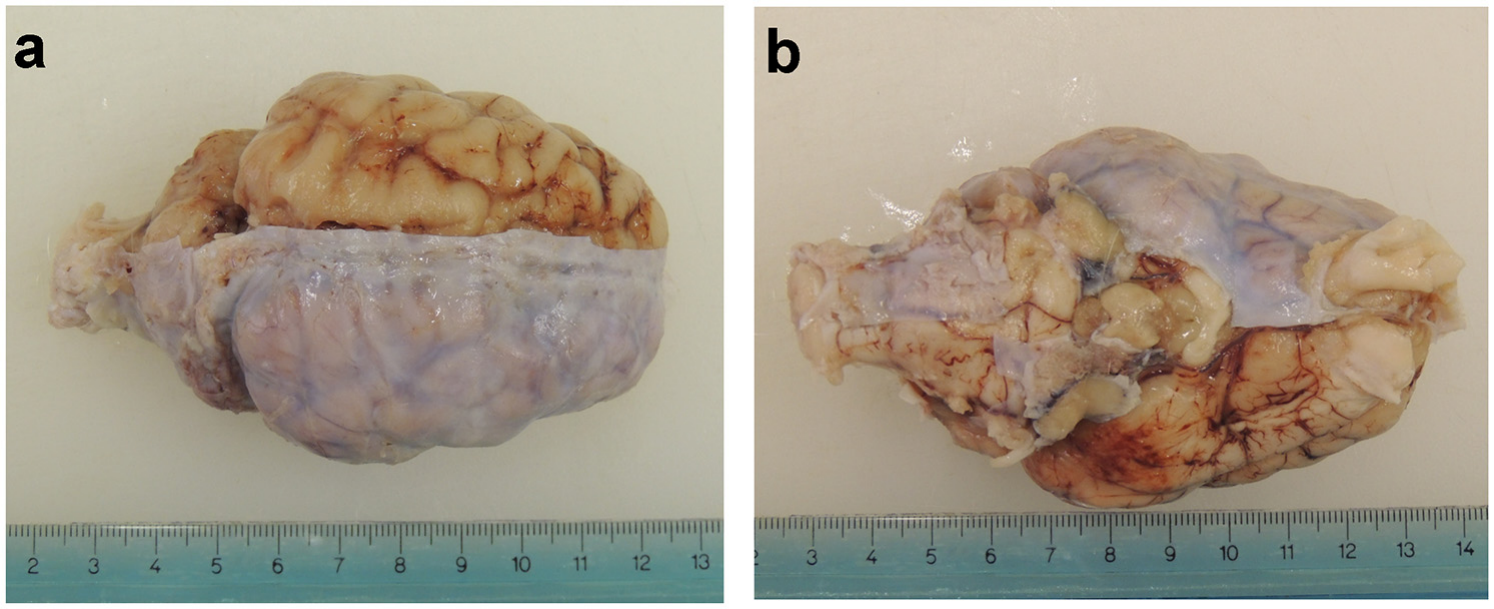
Fixed brain after partial removal of the dura mater. (a) Dorsal view. (b) Ventral view.

The brains were removed and treated according to an established protocol [13]. Briefly, the brains were weighed with a digital precision scale and photographed. The dura mater and the other two meningeal layers were preserved during the extraction of the brain (Fig 1).

After removal, the brains were immersed for 2 months in 4% (w/v) phosphate buffered paraformaldehyde at 4°C to allow hardening and proper fixation. The immersion in paraformaldehyde resulted in an increase in brain weight due to penetration of the fluid. Comparison between fresh and paraformaldehyde-fixed brain weights yielded the following conversion formula: Bw_fresh_ = Bw_fixed_/1,104 where Bw is brain weight. After removal of the dura mater, this meningeal layer and fixed brains were weighed and the following conversion formula was used: Bw_fresh_ = Bw_fixed-dmw_/0,94 where dmw is the dura mater weight. In addition, the weight of the brain and its components (telencephalon, diencephalon, mesencephalon, pons, cerebellum, and myelencephalon) was calculated from fixed brains after careful dissection.

### EQ and CQ

The relationship between the weight of the brain and the body weight to obtain the EQ of each animal, was calculated with the formula EQ = E_i_/0.12P^2/3^, where E_i_ and P are the mean weight of the brain and body, respectively [10]. We kept the value of the exponent 2/3 (or 0.67) originally indicated by [10], although recent studies suggested a higher value (0.75) to better fit mammals with a large body mass (for reference and discussion on this topic see [14,15]. The EQ was calculated using only data from fresh brains.

To calculate the CQ, we used the following equation CQ = Cb_vol_/(0.145M_b_^0.978^), proposed by [16], where Cb_vol_ is the volume of the cerebellum (Cb_vol_ x 1.04 = Cb_mass_ x 0.96) [17] and M_b_ as the brain mass (= brain weight). Since the weight of each brain part was measured from fixed specimens, we applied the conversion formula to determine the weight of fresh tissue (see above).

## Results

### Gross anatomy

The gross anatomy of the swine brain was evaluated only after removal of the dura mater (Fig 1), which was thick and resistant to dissection, as observed by [18].

**Fig 2:**
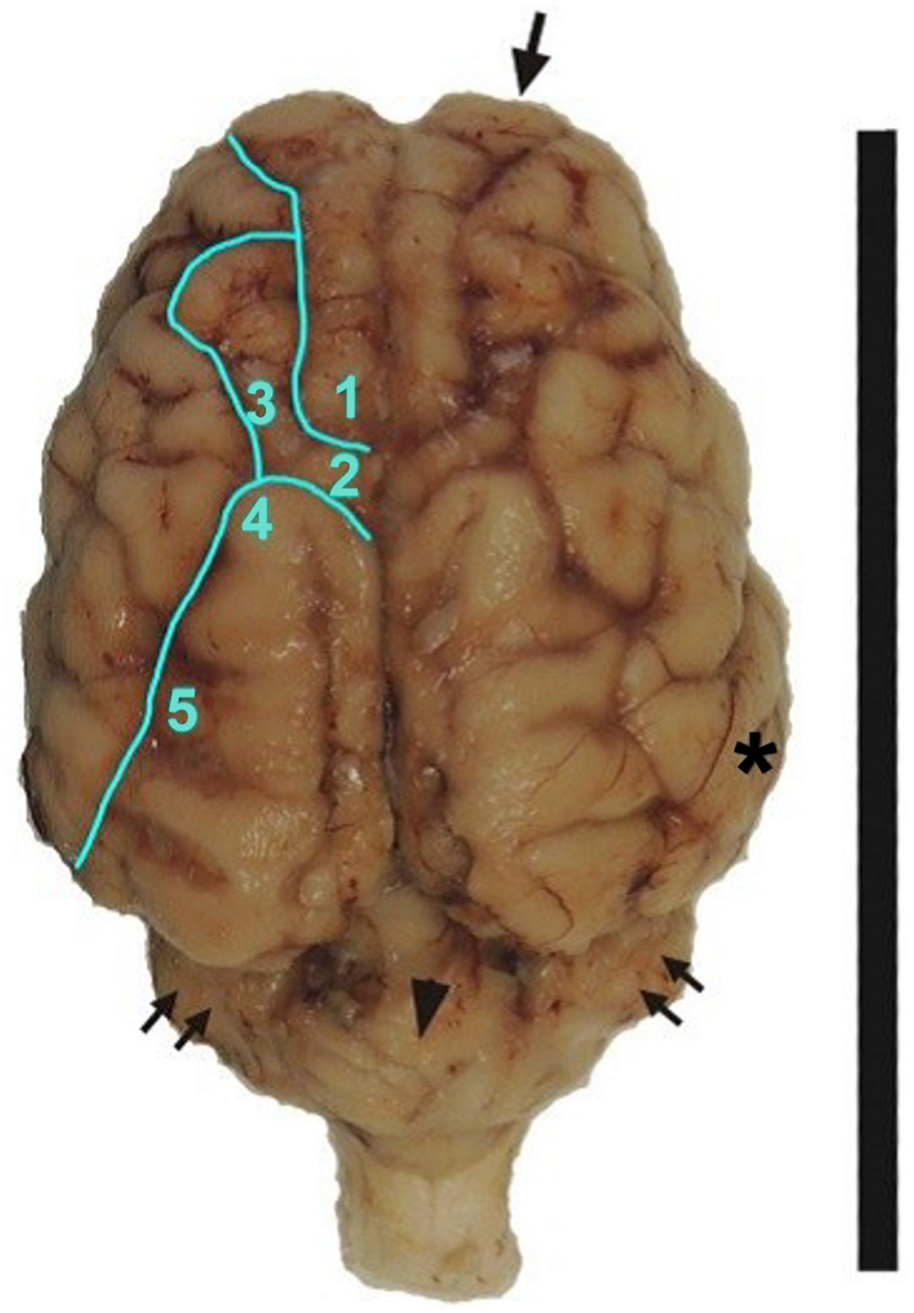
Fixed brain with main sulci shown. 1: cruciate sulcus; 2: ansate sulcus; 3: coronal sulcus; 4: connection sulcus with suprasylvian sulcus; 5: median suprasylvian sulcus. *: temporal lobe. Arrow: olfactory bulb. Double arrows: cerebellar hemispheres. Arrow head: cerebellar vermis. Bar: 10cm.

The dimensions of the newborn brain (4.5 cm wide and 3.5 cm long) are proportionally comparable to those of adults (7.5 of width by 11.5 cm in length). The morphology of the brain recalls overall that of other large domestic ungulates. The olfactory bulbs and cerebellum are visible dorsally and the latter has a rather developed cerebellar vermis compared to the cerebellar hemispheres (Fig 2). The profile of the cerebral hemispheres grows in height in the cranio-caudal axis in a regular curvilinear line. The lateral expansion of the temporal lobe at the level of the inter-insular axis is typical of the Cetartiodactyla. The arrangement of the sulci (Fig 2) follows the general plan of the ungulates [19].

### Fresh brain weight

**Fig 3:**
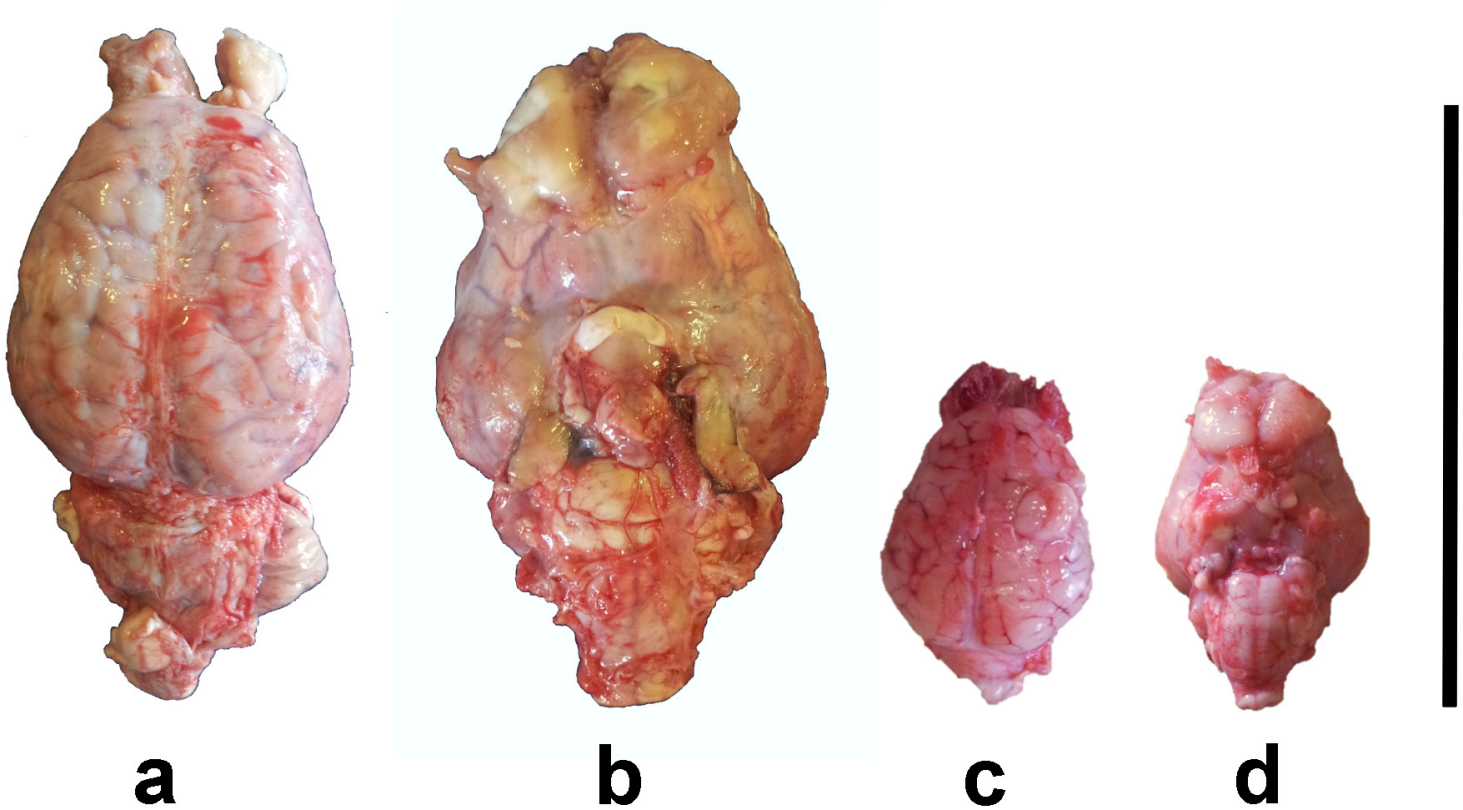
Aspect of the swine brain after extraction. (a) Adult, dorsal view. (b) Adult, ventral view. (c) Neonate, dorsal view. (d) Neonate, ventral view. Bar: 10cm.

The general appearance of the pig's brain after extraction is shown in Fig 3. The fresh weights of the body and brain of all animals are shown in Table 2, while Table 3 shows the average of the weights considered. Since the somatic difference between LW, LR, and LW x LR pigs of comparable age and weight class were minimal, we considered all breeds as a single experimental cohort. We found that fetuses (n = 4) had a mean brain weight of 14,9g (SEM ±1,03) for an average body weight of 150g (SEM ±1). Neonates (n = 13) had a mean brain weight of 32,5g (SEM ±0,6) for a mean body weight of 1,18kg (SEM ±0,075).

**Table 3:** Mean values for body and brain weights of each class of age. SEM: Standard Error Mean.

Age category	n	Body weight (kg) ± SEM	Brain weight (g) ± SEM
Fetuses	4	0,15 ± 0,01	14,9 ± 1,03
Neonates	13	1,18 ± 0,075	32,5 ± 0,6
Sexually mature	29	149 ± 6,69	133 ± 2,29
Young adults	3	77 ± 3	126 ± 6,57
Adults	26	158 ± 5,3	134,5 ± 2,45

### Body weight

The values related to body weight (Tables 2 and 3) were of 0.85 to 1.75kg for newborns (n = 13) with an average value of 1.18kg (SEM ± 0,075). The values for adults (n = 29) ranged from 80 to 200kg. The majority of individuals had a weight comprised between 120 and 180kg. The individuals were divided into two groups on the basis of sexual maturity: young adults (n = 3), in which are included the body weight of individuals of 74–80 kg, and proper adults (n = 26), that is, individuals of 130–200 kg. This division was also respected in the calculation of the weight of the brain and its EQ.

### Weight of fixed brains

**Fig 4:**
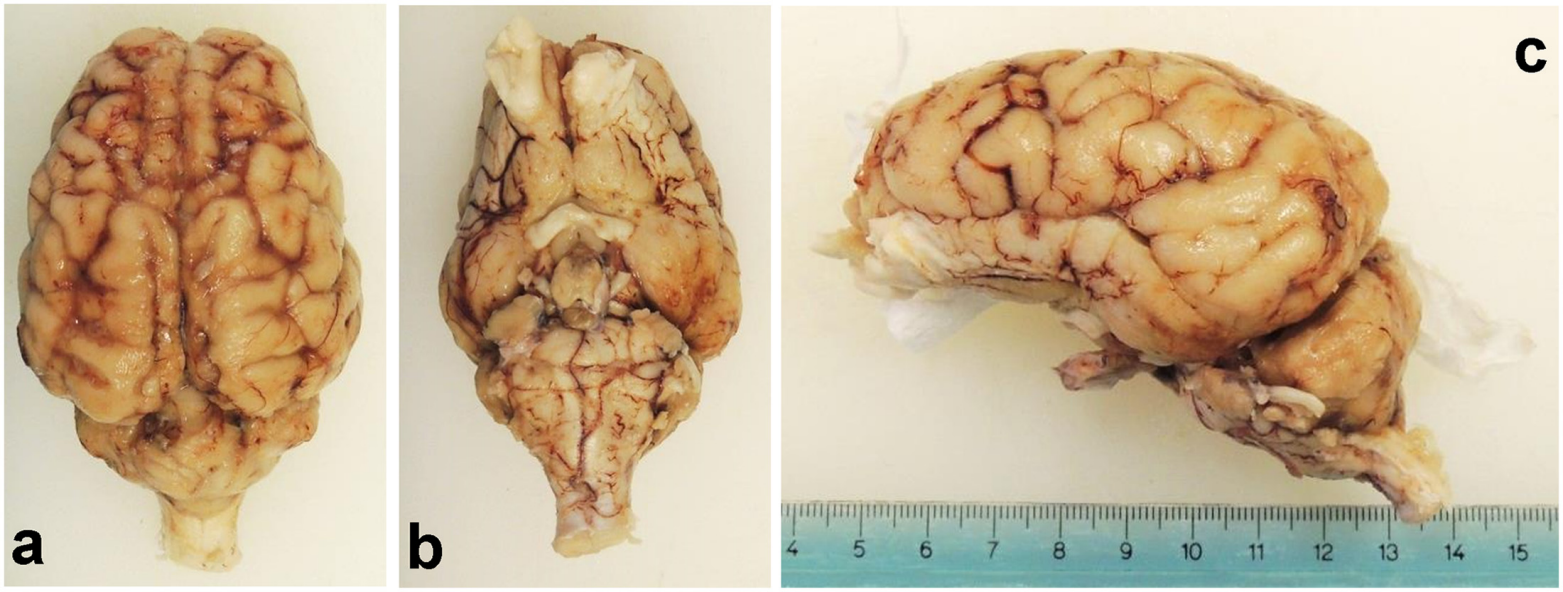
Views of the swine brain after fixation. (a) Dorsal. (b) Ventral. (c) Lateral left.

After successful fixation (Fig 4) the brains were weighed again. The values obtained for the individuals that had reached sexual maturity, representing the majority of the sample, were of 141g (SEM = ± 2.36) in average with a range of 114-168g. More specifically, for the young adult group the minimum and the maximum are respectively 128 and 135g, average of 132g (SEM = ± 3.18), for the adult group of 114 and 168g and 142g (SEM = ± 2.55) on average. The brains of neonates had an average value of 35.1g (SEM = ± 0.43) with a range of 33-37g.

### Weight of the dura mater and brain segments

**Fig 5:**
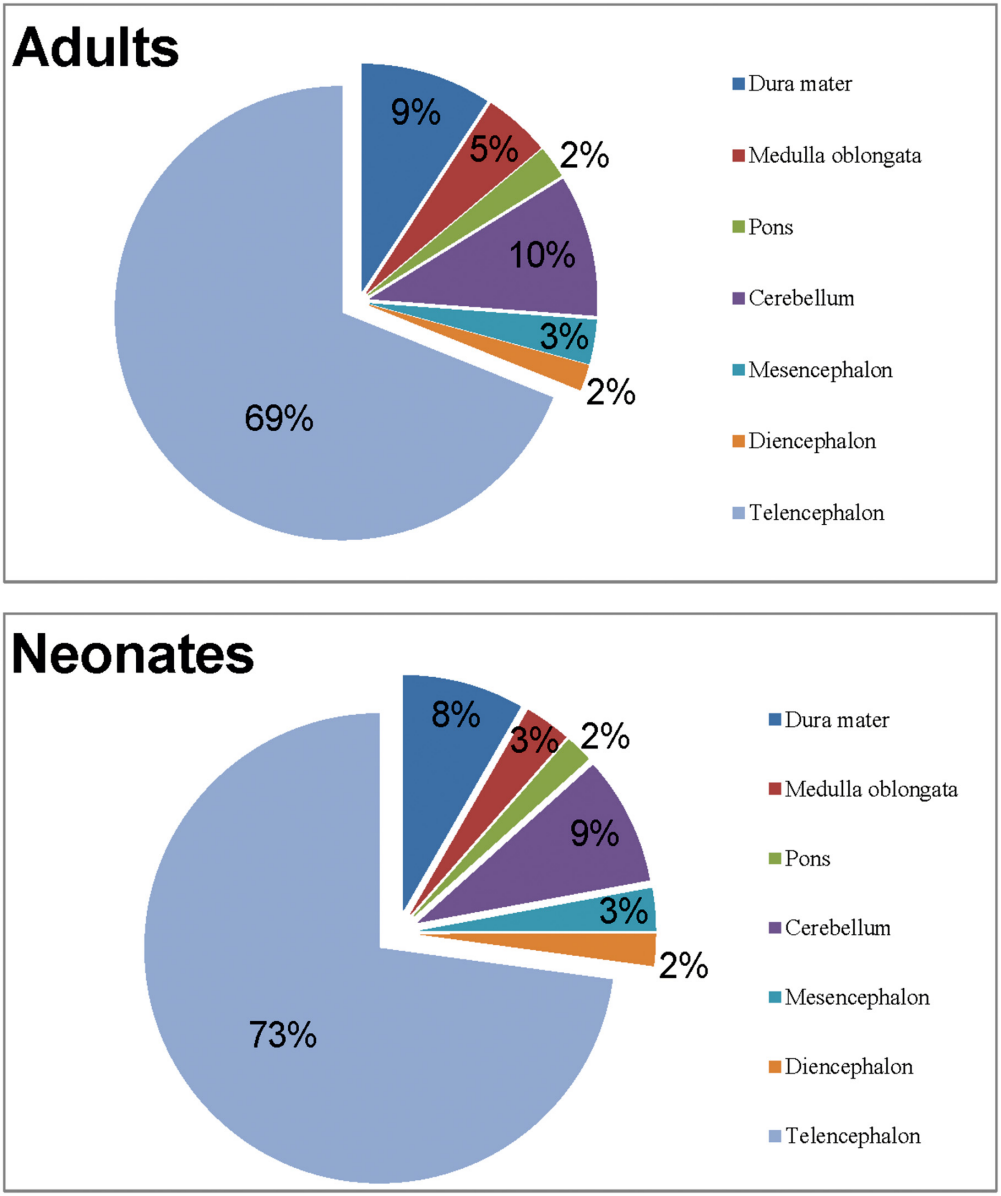
Percentages of the respective weights of the brain parts of adult and neonate swines.

After removal of the dura mater, the main brain parts corresponding to the original neural vesicles were isolated, all structures were weighed (Table 4) and the percentages of the weight of each part compared to whole brain were calculated (Fig 5).

**Table 4:** Mean values of the dura mater and the parts of the brain.

	Dura madre		Medulla oblongata		Pons		Cerebellum		Mesencephalon		Diencephalon		Telencephalon	
	Weight (g)	%	Weight (g)	%	Weight (g)	%	Weight (g)	%	Weight (g)	%	Weight (g)	%	Weight (g)	%
Adults														
Mean	13,12	9,22	6,55	4,66	3,11	2,23	14,06	9,97	4,31	3,10	2,53	1,81	96,52	68,70
SEM	0,58	0,30	0,18	0,11	0,13	0,13	0,29	0,19	0,13	0,12	0,11	0,09	1,58	0,46
Neonates														
Mean	2,89	8,26	1,08	3,14	0,64	1,86	3,01	8,73	0,99	2,86	0,75	2,16	25,01	72,48
SEM	0,30	0,89	0,07	0,20	0,05	0,13	0,07	0,07	0,06	0,17	0,06	0,15	0,44	0,92

#### EQ

**Fig 6:**
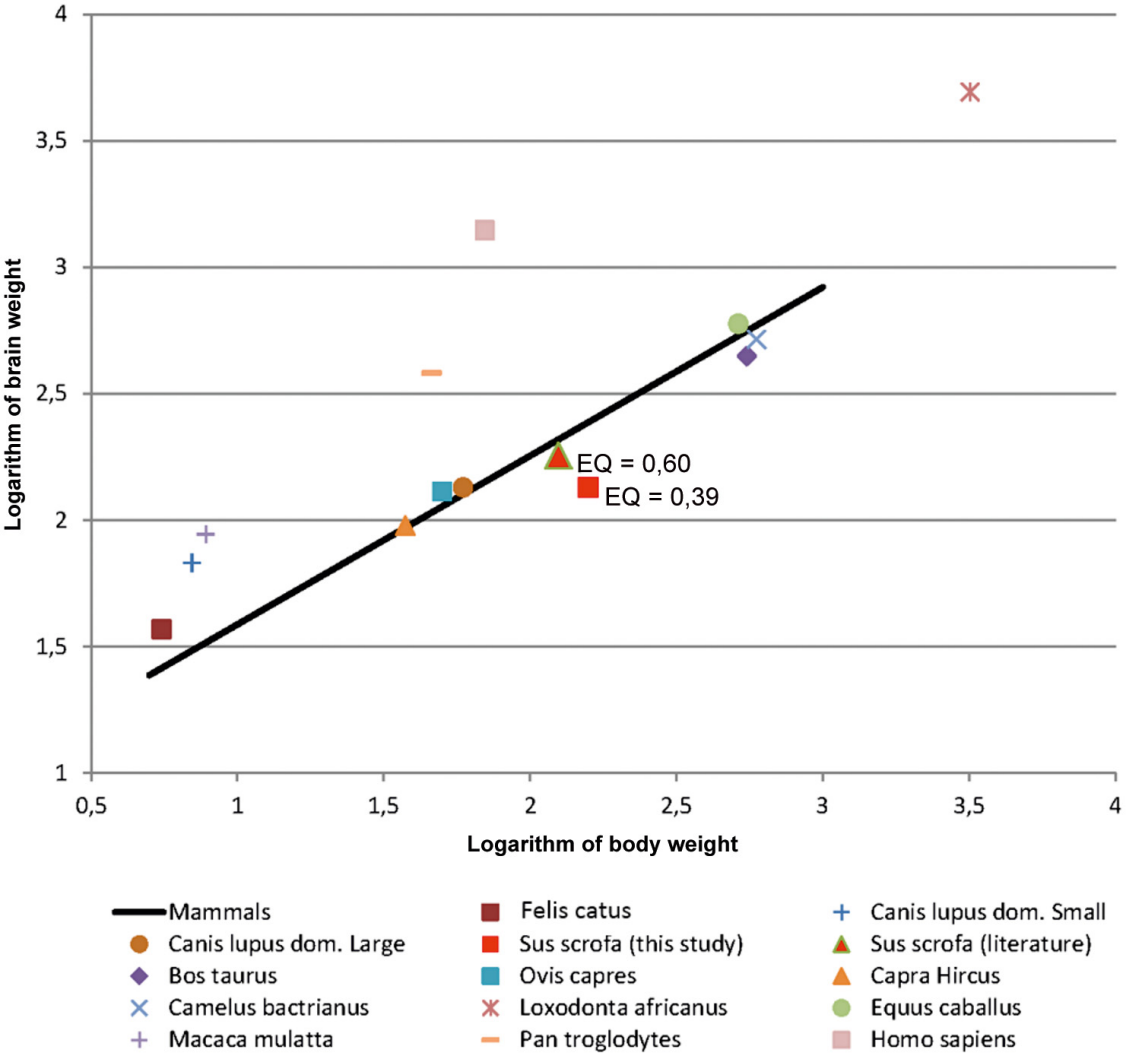
Logarithmic graph showing the evolution of the brain weight in function of the body weight of chosen mammal species. The regression line represents the expected value for the weight of the brain based on the body weight following the equation: Eo = Ei/0,12Pi^2/3^ by [10]. The values above and under the line represent experimental findings with heavier or lighter brain weights per body weight than the theoretical value of the Jerison equation [10].

The EQ of the different groups was as follows: 2.42 for one day piglets (n = 13); 0,58 for young adults (n = 3); 0.38 for adults (n = 26). The EQ of the species obtained by sexually mature subjects (n = 29) is 0.39, and its position, compared to that calculated in other mammals, is shown in Table 5 and Fig 6. A comparison within the adult animals that we used for our study showed that LW adult females (n = 7) have an EQ of 0.34, and LW x LR adult females (n = 12) an EQ of 0.38 (*p* < 0.05).

**Table 5:** Brain mass, body weight and EQ of chosen mammals.

Species	Brain weight (g)	Body weight (kg)	EQ	Source
**Carnivora—Felidae**				
*Felis catus*	37	5,05	1	[10]
**Carnivora—Canidae**				
*Canis lupus familiaris*	68–135	7–59	1,55–0,74	[18]
**Artiodactyla—Suidae**				
Sus scrofa (n = 1)	180	125	0,60	[10]
*Sus scrofa domesticus (n = 29)*	133	149	0,39	This study
**Artiodactyla—Bovidae**				
*Bos taurus*	445	550	0,55	[18]
*Ovis aries*	130	50	0,80	
*Capra hircus*	95	37,5	0,71	[37]
**Artiodactyla—Camelidae**				
*Camelus bactrianus*	518	594	0,61	[38,39]
**Proboscidea—Elephantidae**				
*Loxodonta africana*	4927	3185	1,67	[52]
**Perissodactyla—Equidae**				
*Equus caballus*	599	514	0,78	[13]
**Primates—Cercopithecidae**				
*Macaca mulatta*	88	7,8	1,86	[10]
**Primates—Hominidae**				
*Pan troglodytes*	382	46	2,48	[10]
*Homo sapiens*	1300–1400	70	6,62	[62]

#### CQ

Like the EQ, the CQ varied considerably in the different groups studied. The values were of 0,59 in infants; 0,62 in young adults; 0,71 in adults and 0,7 in sexually mature animals.

## Discussion

The present study, based on a statistically significant sample of 46 brains, provides a macroscopic gross anatomical description of the brain of the domestic pig *Sus scrofa domesticus*, the absolute and relative weight of its parts, the calculation of the EQ and CQ values, as well as a position relative to other mammals.

The first striking aspect of the brain of the domestic pig is its rather small size compared to the mass of the animal. Remarkably, the average adult brain weight fluctuated between 107 and 160g. This gap in absolute weight is reasonable when considering the heterogeneity of individual weights in the sample (adults weight from 70–80 kg up to 200 kg). This marked gap in addition to differences attributable to race, is due to the typical Italian pig farming system distinguishing two categories of pigs. The first is that of light-weight pigs (80–100 kg of live weight) intended for consumption as fresh meat, the second is that of heavy-weight pigs that reach up to 200 kg and are used for the production of sausages. So, the tremendous increase in body weight imposed by industrial farming condition certainly influences brain-to-body weight measures, including the EQ.

The weight of the brain at birth averaged 32,5g with a body weight of 1,18kg. As expected, the remarkable brain size for a neonate is due to the particular mode of replication of neurons which increase in number during the earlier fetal stages and, to a much lesser extent after birth, with the exception of the olfactory bulb. As for the body weight, the fluctuation range it is to be considered absolutely normal even if the maximum value is almost double the minimum given the relative size of the piglets depends on the number of births per sow and the number of brood [20].

Different methodologies, together with differences and variability between age classes of the specimens considered, may lead to different results: The data obtained in this study do not agree with another report including the same species in which the authors described a higher brain weight and a lower body mass [21].

In several publications consulted for the present study, the Authors do not specify how the brain weights were obtained: fresh or after fixation; after how many hours *post mortem* (if fresh); if the dura mater was included or excluded in the data disclosed [21–25]. The experimental series was often reduced in numbers, old, or derived from various studies involving different sampling methods (including the use of cranial measurements to derive the volume of brain perfusion and fixation) or unspecified methodology [23]. There is also a frequent use of database (without support of new samplings) to increase the sample size [15,21,25,26,27]. A detailed comparison between the data obtained in this study and data available is therefore made rather difficult (see [28] for discussion and criticisms).

It is widely believed that the brain has suffered a progressive increase in size over the course of evolution [10,21,24,26,27,29,30] with the consequence, more or less explicit, that at each increase of magnitude corresponds an increase in function. Learning skills, foraging strategies, habitat management capabilities have been variously linked with this aspect, and sometimes considered the primary cause. To date, the most likely hypothesis remains that the social brain (SBH, Social Brain Hypothesis) developed in primates [30], but is also applicable in varying degrees to carnivores [24] and artiodactyls [21]. It is based on the principle that in complex social groups, such as those of primates, or gregarious animals as can be ungulates, develop relational dynamics that often require the ability to manage individual conflicts and the need to remain in the group, thus the need to cope with huge computational demand, contributing to the progressive increase of brain size [21,26,27]. The role of a greater or lesser magnitude of the brain is still a topic widely debated. The brain is one of the organs requiring the most energy (preceded only by the heart) and the cost of its operation is about 8–10 times higher per mass unit than that of skeletal muscles. Evolution is an ''economical process'' and does not enlarge an organ from which the body cannot derive a real benefit [29]. In mammals the increase in size is due predominantly to the enlargement of the forebrain and neocortex, leading to an increase of the primary areas (receiving thalamic afferents: motor area, visual, auditory, somatosensory) as well as secondary ones (higher mental functions). Progressive specialization and diversification in the mammalian brain arises precisely from the differential development of these areas, and the possibility to adopt flexible behaviors. To balance this line of thought, there is the paradox of the miniaturized brain of insects. These animals are still capable of very complex (but relatively stereotyped) social behavior and have considerable spatial and visual skills. It seems therefore that ''basic functions'' can be allocated in very small spaces [31].

Studies on brain size may group species of different orders and apply a number of indexes to assess the evolutionary position of each group. The EQ represents how many times the brain is larger (or smaller) than what would be expected for a given species relative to its body size [15], and encephalization has been considered to correlate with improved cognitive abilities across species and even intelligence [32]. In some species brain mass significantly differs from the expected one in a way that has been suggested to be functionally significant. A great deal of evidence implies that larger EQs or relative brain size endow species with improved cognitive abilities [33], behavioral flexibility, such as the ability to respond successfully to novel environments [34] or to alternate between feeding strategies [35]. These findings seem to agree with the fact that humans, dolphins, and chimpanzees have the largest known EQs [36]. The EQ of Ungulates differs among the species ranging from 0.91 to 0.78 in Perissodactyla (*Equus caballus*) [13,27], to 0.55–0.80 in Cetartiodactyla such as Bovidae [18,37] and Camelidae (*Camelus bactrianus*) [38,39]. In Suidae, it has been reported that the EQ of only one specimen of *Sus scrofa* (un-indicated subspecies) was 0.60 [27] (Fig 6).

The average value of 0,39 obtained in this study for the domestic pig with differences between young adults (0,58) and adults (0,38), is significantly less than 1. Such a low index nevertheless falls within the wide range indicated for Cetartiodactyla (0,14 to 4,43) compared to primates and Carnivora. At the top of the range are the odontocetes (average EQ = 3,10), alone sufficient to raise the upper limit of the range, while the terrestrial Cetartiodactyla are at much lower values [15]. Even compared to cattle, sheep and goat, the pig stands in a rather lower position.

Various reasons are responsible for a low EQ value, including the existence of very heavy animals reaching 200 kg, a strong domestication pressure [40,41], and the intrinsic nature of the Jerison model [10] which may be unfavorable for species of large size. To this effect we also note that within our samples, heavier LW sows had a lower EQ (0.34) than cross-bred LW x LR (0.38), emphasizing the direct effect of body weight (see below for further discussion).

The evolutionary pressure can alter in at least two ways the brain/body size ratio. It could directly affect brain size or alternatively be a consequence of increase or decrease of the body size [21]. In the case of domestic mammals bred for the purpose of food production, the selective pressure is directed towards the increase of body weight in order to obtain a higher yield at slaughter, affecting the EQ to a lower value. Kruska [40,41] also showed that bred animals have an absolute brain weight lower, and therefore a lower EQ compared to wild progenitors. Domestication is perhaps the longest and most important experiment in genetic selection, and involved especially Lagomorphs, Cetartiodactyla, Perissodactyla and Carnivores. This process in all cases led to a reduction in absolute brain size. The quantification of this reduction depends on the single species. In fact, species with originally low encephalization (i.e. Lagomorphs) showed a smaller decrease than Carnivores. In the case of the domestic pig we noted that the brain weight decreased by 34% compared to the wild progenitor. This fact alone is sufficient to explain the EQ found in this work.

The brain and body values reported for *Sus scrofa* by Shultz and Dunbar [27] would produce an EQ = 0,6 for the pig, which is higher than the value reported here. The measures attributed to *Sus scrofa* in their work are of 180g of brain weight (size in the original Table of [27]) for 125kg of body weight. Given the relatively large brain weight which was never matched in the sample considered in this study, and the rather reduced body size reported in [27], it is possible that the authors referred to the wild boar and not to the domestic swine. The given name of *Sus scrofa* and not of *Sus scrofa domesticus* suggests this eventuality. An alternative explanation could be the different breed considered or the origin/destination of use. In the first case if the animal had belonged to a rustic breed, rather than to one commonly used for meat production, it could have retained ancestral characteristics, including a reduced body size with a relatively heavy brain. In this regard, it is logical to ask what impact can obtain the reduction of the mass of the brain following the process of domestication [40,41]. It would be logical to expect that the decrease is associated with a reduction in functional capacity [40,41]. Studies explicitly based on wild specimens are extremely rare to come by, as the domestic and wild pig basically belong to the same genus and—depending on the taxonomy—to the same species. A study centered on the pigmy hog (*Porcula salvanius*) indicates a similar level of encephalization for several members of the Suidae family, except *Sus scrofa* that maintains a larger brain [42]. A 16% reduction in brain size due to domestication has been reported also in domestic Perissodactyla [43]. Surprisingly, despite a 20–30% loss of brain mass relative to wild ancestors a ''domestic brain'' is still able to deal with wildlife if the individual is reinserted in natural context. From what emerges from experimental tests [44] in rats and dogs [45] these animals are even faster learners compared to wild animals. It appears that functional capabilities are preserved despite reduced size hence it seems appropriate to consider these changes as a form of adaptation to a particular ecological niche. While the body size alone remains the best predictor of the mass of the brain, we cannot say that the mass of the brain is a predictor for the behavioral repertoire and cognitive abilities [31].

The apparent high EQ of piglets (EQ = 2,42) and its subsequent decrease is explained by the lack of body fat at birth, by the advanced maturity of the central nervous system at birth. Pigs belong to a precocial species, as well as horses and cattle. A few minutes after birth they must be able to stand and move independently, a notable difference from altricial offspring. This is further shown by “multiplication factor”, the number by which the brain size of the newborn must be multiplied to obtain the corresponding value of the adult. In precocial offspring species this number is between 1–6, while for those with altricial offspring it is 7–12 times. In the case of the pig, brain mass will increase by about 4 times while the body size (fat and muscle) will grow by 60–70 times. Somatic development occurs primarily after birth while brain growth is biphasic. The first phase sees a very rapid growth of the brain, while in the second phase growth is slower than the rest of the body and the brain reaches maturity before complete somatic development. This brain growth pattern is common to all mammal species with the due differences: in precocial species the first phase takes place during fetal development while in altricial species the first phase is carried out immediately after birth [40,41]. Therefore, mammal brain size differences are to be found mainly in embryogenesis and ontogenesis processes [21,40] and thus explain the relatively higher EQ in young individuals than adults. An important collection of data on the macroscopic anatomy of the piglet brain can be found in the online collection of University of Illinois (http://pigmri.illinois.edu/).

Consistently with evolutionary phylogeny showing that the encephalization increase is largely due to telencephalization (increased cerebrum) [40,41], our analysis of the weight of each brain part shows that the cerebrum alone occupies 70% of the entire brain. The neocortex gradually increases in size taking alongside its peculiar stratification pattern characteristic of mammals. Finlay and Darlington [46] detected an apparent regularity in the sizes of mammalian brain structures and the mass of belonging brains. These authors demonstrated this consistency in size of the cerebellum relative to total brain mass, cerebellum quotient, when examining a series of insectivore and primate brains. However, to date exceptions to this allometric regularity have been documented for cerebellar size in relation to brain mass of microchiropteran, odontocete cetacean, and African elephants’ brains [12,47,48]. Our results indicate that in the pig the second heaviest part of the brain is the cerebellum with a CQ of 0,7. Macroscopically the cerebellar vermis and hemispheres appear of the same size, an aspect described also by [49] in the African elephant, for which they indicated a volume of 4,47 and 4,81 ml respectively. The development and relative expansion of the cerebellum is a parameter indicative of the general motor skills of a given species, and reflects the general capabilities in the regulation of body posture and movement coordination. In particular, the large size of the archicerebellum, including the connections to the lateral vestibular nucleus and the vestibulospinal tract, are justified by the function of rapid connection center for quadrupedal movement [40,41,49,50]. This CQ is in the same range as the bovine (0,725) [51], but falls short of half the value for elephants (1,66–1,84) and primates (0,71–1,28) [12]. Additional considerations about the CQ value are difficult to express because they lack elements of comparison and literature on the specific topic is almost non-existent. The remaining parts seem to follow the proportions given by other authors for ungulates [13,37].

The domestic pig shows a convoluted brain characteristic of Cetartiodactyla with pronounced individual variations. One general feature of the order is the lateral extension of the temporal lobes [18]. This expansion, noteworthy in cetaceans, is not so evident in pigs compared to other terrestrial members of the order (e.g. cattle) and could show evolutionary convergence towards auditory sensitivity with different degrees according to family- and species-specific developmental trends. The design of the grooves has significant individual variations (e.g. differences between hemispheres) despite species-specific characteristics [18]. In mammals the number and width of the sulci (primary and secondary) also seem to depend on the somatic mass as well as the phylogenetic position (Proboscidata have an extremely convoluted brain) [49,52–55]. In mammals of veterinary interest while the cow and the horse have a very complex sulci arrangement with tortuosity, the pig is placed in an intermediate position with a more linear topography, unlike carnivores (dogs and cats) which show a more simple anteroposterior parallel organization.

The different sulci and gyri were definitively mapped in the man and some laboratory animals, for which stereotactic atlases are available. We tried to derive by analogy the topography in other species. The situation is further complicated by the fact that homologous convolutions should match not only for position and course but also in the cytoarchitectural structure, which is realized only in part [18]. Comparative studies have shown that the neocortex varies regarding the arrangement of the areas [13]. Based on this comparison, ten “main” sulci always recognizable and other “accessory” sulci subject to greater variability have been identified in ungulates [19,54]. In this study, the grooves nomenclature follows the NAV, but alternative names are also available in literature.

In conclusion, our data suggest that correlations between brain size and complex behaviors remain unclear, at least in the pig, and while EQ and CQ comparative measurements shed light on the position of *Sus scrofa domesticus* among other domestic and wild mammals, the relative weights and lobe developments could be related to feeding behaviors, environment and social adaptations or sensorial specialization [28]. The data proposed in this paper contributes to better characterize the poorly understood brain capabilities of a domestic species very common in the world, as public concerns regarding animal wellbeing and living conditions rise. Being separated from its wild ancestor by human heavy selection for centuries may reduce scientific interest towards the domestic pig, but the low inter-individual variability is an aspect looked for in laboratory animals, also heavily selected and relied upon for the immense majority of scientific work. The use of domestic mammals could then be a valuable alternative and preserve laboratory animal lives [56], as recommended by several neuroscience societies and the European Community regulations.
